# What Parents Say and do: Parental Responses to Asian American Young Adult Mental Health and Help Seeking

**DOI:** 10.1007/s10826-026-03259-4

**Published:** 2026-01-29

**Authors:** Miwa Yasui, Eunseok Jeong

**Affiliations:** Crown Family School of Social Work, Policy, and Practice, University of Chicago, 969 E. 60th St, Chicago, IL 60637 USA

**Keywords:** Mental health socialization, Young adults, Asian american, Parent socialization, Mental health

## Abstract

Asian American cultures may hold culturally construed views of mental health and help-seeking that are contrary to Western, biomedical notions of mental health and treatment. Asian American young adults may learn culturally specific views of mental health and help-seeking through the family, which is the primary socialization context for children. Parental mental health socialization, a central mechanism through which parents transmit messages about mental health and help-seeking to children, is designed to shape the way Asian American young adults understand and respond to distress. This study developed and tested the Parental Mental Health Socialization – Parental Responses (PMHS-PR) scale, one of the first measures of parental processes of mental health socialization among a sample of 486 Asian American young adults. Using a split sample approach, exploratory factor analysis first determined the factor structure of the measure on Sample 1 (*n* = 243), followed by a confirmatory factor analysis on Sample 2 (*n* = 243). Correlations between derived factors with measures of stigma attitudes and mental health outcomes were examined. Results revealed a bifactor structure with one general factor of parental mental health socialization and five specific factors: Parental Stigma Toward Youth, Parental Supportiveness, Enduring and Overcoming Distress, Hiding Mental Health from Others, and Parental Silence. The PMHS-PR showed good internal consistency reliability and concurrent validity with depression and somatic symptoms and stigma attitudes towards mental illness. Findings indicate that the PMHS-PR is a multidimensional, psychometrically sound scale that measures broadband and narrowband dimensions of parent mental health socialization among Asian Americans that are linked to salient young adult mental health outcomes. This signals its importance in identifying familial influences that centrally shape mental health attitudes and help-seeking among Asian American young adults.

**Supplementary Information:**

The online version contains supplementary material available at 10.1007/s10826-026-03259-4.

## Highlights

Parental mental health socialization is a key mechanism through which Asian American children learn and develop an understanding of mental health and help seeking.

The new empirical measure of parental mental health socialization captures culturally anchored parental responses to youth mental distress.

Parental messages of cultural mental health stigma, parental silence, enduring and overcoming distress and hiding distress is related to Asian American young adult mental health and stigma attitudes.

Finding highlight the crucial role of parental socialization in shaping Asian American children’s view of mental health and openness to mental health.

**Supplementary Information:**

The online version contains supplementary material available at 10.1007/s10826-026-03259-4.

As the fastest-growing racial group in the United States, Asian Americans are projected to become the fourth largest racial group by 2060 (U.S. Census Bureau, 2016). In the U.S., Asian Americans generally referred to individuals whose origins are from the Far East, Southeast, or South Asia, and the largest ethnic groups include Chinese, Indian, Filipino, Vietnamese, Korean, Japanese, Pakistani, Cambodian, Hmong, Thai, Lao, Bangladeshi, Burmese, Nepalese, and Indonesian (Pew Research Center, 2021; Census Bureau, 2016). While heterogenous in country of origin, language, and culture, Asian Americans represent a racial group that is touted for its academic and economic success. This has resulted in Asian Americans being often referred to as the “model minority,” which describes them as the successful, “problem-free,” law-abiding ethnic minority that has endured and overcome hardships, including racial discrimination and oppression, through hard work and good moral values (Shih et al., 2019; Suzuki, 2002).

Yet contrary to this “model minority” stereotype, Asian American youths report higher levels of depression and emotional distress when compared to their European American counterparts (Chen et al., 2019). National data from the Healthy Minds Study indicated that 20% of Asian American students reported moderate to severe depression (Lipson et al., 2022). Young adult Asian Americans between the ages of 18 to 25 have the highest rates of having serious thoughts of suicide (9.8%) compared to other racial and ethnic minority groups (SAMHSA, 2019). On university campuses, Asian American youths are reported to have the highest proportion of suicide deaths (Leong et al., 2007). Asian American college students are also reported to have higher levels of self-injury, suicidal ideation, and suicide attempts than their White, Black, and Latinx peers (Liu et al., 2019). These statistics are sobering, considering the additional sociocultural stressors that Asian American youths face including acculturation, intergenerational conflict, and discrimination (Fuligni, 1998).

Despite these mental health needs, Asian American youth and their families are the least likely, of all racial and ethnic groups, to seek mental health care (Garland et al., 2005; Lau et al., 2011). Yang et al. (2020) found that 78% of Asian Americans with serious psychological distress (SPD) and 73% of those experiencing a major depressive episode (MDE) in the past year did not receive mental health care compared to 51% of Whites with SPD and 42% with MDE. Lipson et al. (2022) examined mental health prevalence and service use rates across U.S. college students from 2013 to 2021 and found that Asian American students persistently underutilized mental health services compared to other races. Among those experiencing psychological distress, Non-Hispanic Whites were twice as likely to use mental health services compared to Filipino Americans and 3 times as likely than Asian Indian and Chinese Americans (Balaraman et al., 2023).

Several underlying mechanisms of this underutilization exist, including logistical barriers, mental health stigma, low mental health literacy, and cultural incongruence (Garland et al., 2005; Lau et al., 2011; Yasui et al., 2017). Mental health literacy, described as having knowledge of and recognition of mental health disorders, as well as knowledge of treatment and coping options (Jorm, 2012), has significant implications for service utilization. Evidence suggests that higher levels of mental health literacy is associated with positive mental health outlooks (Beatie et al., 2016), whereas lower mental health literacy is linked to negative attitudes towards mental health services, delayed help seeking and poor treatment engagement (Johnston & Freeman, 2002). Compared to Whites, Asian immigrant populations are in general, less likely to identify distress as a mental health disorder and apply corresponding terminology (Kalibatseva et al., 2014), have less knowledge of mental health service access and use, and are less willing to seek professional mental health help (Leong & Lau, 2001). Moreover, studies note that cultural beliefs about mental health contribute to alternative views about the cause of mental health problems (Yasui et al., 2017) and appropriate sources of help (e.g., use of herbal medicines, traditional or spiritual healers; Leong & Lau, 2001; Yasui et al., 2017). Thus, Asian American youth may learn about mental health and approaches to healing from culturally informed pathways that differ from MHL, which follows a Western, biomedical understanding of mental illness (Jorm, 2012).

In fact, evidence suggests that contrary to Western notions of mental health, Asian American cultures hold culturally anchored views of wellness that perceive wellness as holistic, integrating the body, spirit, mind and human relationships (Fung & Wong, 2007). For East and Southeast Asians, cultural virtues of conformity to norms, filial piety, emotional and behavioral control that are found in Confucianism influence the attributions of emotional distress to character weakness, immaturity, lack of willpower, or self -control (Rhi, 1986; Park et al., 2010). In parental socialization processes, parents discourage children from expressing their emotions or personal vulnerabilities and instead, encourage them to express distress through somatic forms of communication (Lin et al., 1982). Moreover, family members are encouraged to refrain from expressing strong emotions or disclosing personal problems so as to avoid interpersonal conflict or burdening others (Yeh et al., 2006).

## Parent Mental Health Socialization

Parent mental health socialization, defined as the “socialization of mental health beliefs and approaches to healing” from parents to children (Yasui et al., 2023 ) is a salient mechanism that underlies how youths come to develop beliefs, attitudes, and behaviors regarding mental health and help-seeking. It is a bidirectional process that involves (a) parental messages on mental health to youth and (b) youth receipt and interpretation of these parental messages. Parental mental health socialization may serve a primary mechanism that underlies the divergent understanding of mental health among Asian American youths and their families. The family context is considered central in shaping children’s values, beliefs and practices regarding emotion (Eisenberg et al., 1996, 1998), race and culture (Hughes et al., 2006), and mental health (Yasui et al., 2023). The impact of parental mental health socialization may be more pronounced for Asian American families, given the cultural emphasis on interdependence, which may incline Asian American children to be strongly culturally bound to their parents and hence, prioritize familial expectations and goals (Kim, 2007; Tsai et al., 2020).

What Asian American parents believe about mental health and how they respond to their children’s distress will presumably influence how Asian American children experience mental health and seek help for it. In support of this, Yasui et al. (2023), found that parental messages to youth on mental health consisted of those regarding the parental beliefs about mental health (i.e., conceptualization of mental health) and parental responses to mental health, which include how parents respond to their (a) own mental health and (b) youth mental health. These socialization pathways parallel those identified in the emotion socialization literature, which include parents’ beliefs about children’s emotions, parents’ reactions to their children’s emotions, and parents’ own expression of emotion (Eisenberg et al., 1998). Parental mental health socialization pathways are perceived as dynamic and interactive – thus, what parents believe about mental health will likely direct how they respond to their child’s mental distress, and consequently, shape children’s own beliefs and responses to distress.

## Parental Beliefs about Mental Health

What parents believe about their child’s mental health is crucial to the identification of mental health problems and approaches to seeking help. Evidence suggests that when parents recognize their child’s problem as a mental health issue, they are more likely to engage in help-seeking — for example, Brown et al. (2014) found that parents who believed that their child’s mental health problems were not as severe tended to delay help-seeking. Parents may conceptualize their child’s emotional and mental distress as normative (e.g., teenage behavior) or interpret it as defiance or rebelliousness and downplay the severity of the problem, which can result in further delays in help seeking (Moses, 2011).

Cultural variation in parental beliefs about mental health indicates that parents of color are more likely to have a higher threshold for determining whether their child’s distress is a problem and more likely to delay seeking professional help for their children compared to White parents (Bussing et al., 2003; Ho et al., 2007). This may be attributed to the varied conceptualizations of the child’s distress; compared to Caucasian parents, parents of color were less likely to view their child’s problems as a psychiatric problem and were more likely to provide alternate explanations for their child’s distress such as acculturative stress, academic pressure or developmental changes (Cauce et al., 2002; Thompson & May, 2006).

Parental stigma towards mental illness is also associated with negative parental responses and poor engagement in mental health services. Johnco and Rapee (2018) reported that parents who endorsed stigmatized views about depression were more likely to be critical of their children’s symptoms of depression and less likely to be supportive compared to parents who had less stigmatized views. Turner et al. (2015) noted that stigma attitudes towards seeking mental health assistance was linked to lower intentions to seek help in the case of Hispanic parents, but not for African American or European American families, suggesting cultural differences at play.

Among Asian Americans, mental illness is generally viewed as shameful and counter-cultural (Lee et al., 2009), the perceived causes of which are attributed to moral failure, character defect, or biological deficits (Hsiao et al., 2006). Such stigmatized views produce psychological and social effects that have significant implications for individuals, including increased discrimination due to mental illness and elevated depression, anxiety, and thoughts of suicide (Lee et al., 2009). Furthermore, familial and societal responses to individuals with mental health struggles tend to be discouraging. For example, among Asian American parents, stigma associated with a child’s mental health problems is extended to the family, resulting in fear or loss of face, which is described as the risk of losing one’s reputation, honor, prestige, and social value in salient social contexts such as family and community (Kramer et al., 2002). Asian Americans are also more likely to be discouraged from befriending, dating, marrying, or having children with people with mental illness (Yang et al., 2007). The fear of shame or “losing face” associated with mental health stigma can result in increased delays in seeking treatment (Lee et al., 2009).

The implications of stigma may be even more pronounced for Asian Americans because their cultures are more collectivistic and interdependent (Yeh et al., 2004). Because Asian cultures tend to uphold the family over the individual, familial worldviews and goals are prioritized over individual needs, and the successes and failures of individual family members are considered to reflect the family as a whole. Therefore, when a family member has mental illness, it is often considered to be the whole family’s problem (Sanchez & Gaw, 2007). The family’s views about mental health play an instrumental role in how the individual makes sense of their mental health struggle and approaches to help-seeking. As a result, individual decisions regarding the seeking of mental health assistance are often made within the context of the family, with elders and parents holding an authoritarian role. This parental involvement, however, may deter Asian American young adults from disclosing their mental health problems to their family, due to fears of stigma.

## How Parents Respond To their Children’s Mental Distress in the Familial Context

To date, few studies have directly examined how parents *respond* to their children’s mental distress. The literature on emotion socialization provides a helpful framework for examining mechanisms of parental socialization and how these may relate to children’s mental health and help seeking behaviors. Emotion socialization consists of what parents believe and how they respond to children’s emotions, as well as how parents themselves express emotion (Eisenberg et al., 1998). When parents respond punitively or minimize the negative emotions of their children, the latter’s socio-emotional development is adversely affected, whereas supportive parental responses are related to socio-emotional competence (Fabes et al., 2002). However, cross-cultural variations remain — for example, in a study on Nepali families, Cole and colleagues (Cole & Tamang, 1998, Cole et al., 2006) found that Tamang parents would minimize or punish their children’s expression of anger, whereas Brahman parents would respond to their children’s anger with reasoning or by attending to their demands. The authors noted that the variation in socialization of anger within Nepali families may reflect differing cultural values and social histories – for the Tamang, anger is likely to be seen as the hindering of competence in children which is associated with being socially skillful, and promoting positive feelings in others, while for the Brahman, anger may serve an important function in preserving their high caste status, which may reduce parental efforts to minimize children’s expressions of anger. Saw and Okazaki (2010) found that the suppression of emotion was encouraged or valued among most Asian American college students, but only for about half of the White American college students. Among only Asian American students, parental encouragement of suppressing emotion was linked to social anxiety but not depression. Le et al. (2002) found that higher parental display of physical affection and lower avoidance in response to negative emotions were associated with better abilities in identifying emotions among children. Moreover, these relations differed for Asian American and European American young adults. Doan and Wang (2010) reported that compared to Chinese immigrant mothers, European American mothers referenced more thoughts and emotions, whereas Chinese immigrant mothers were more likely to reference behaviors, suggesting varied socialization patterns by culture.

Studies on mental health suggest that family members may respond with stigmatized attitudes when they perceive the distress as a mental illness. For example, Lee et al. (2009) found that Chinese families reported despising or disliking the family member with mental illness and were inclined to hide them from society. Sun et al. (2014) reported that family members of individuals with mental illness in China had a greater preference for social distance compared to the general public, highlighting the impact of stigma within the family. Among Chinese immigrant families, youth reported that parents responded to youth expressions of distress in a variety of ways including dismissal, minimization, supportiveness, or endorsement of culturally anchored approaches (Yasui, 2017; Yasui et al., 2023 ).

## How Parental Beliefs and Responses Affect Children’s Mental Health and Attitudes Towards Mental Illness

Given the aforementioned literature, what parents believe and how they respond to their children’s mental health play a salient role in children’s mental health outcomes and help-seeking behaviors. González-Torres et al. (2007) found that family members of patients with schizophrenia responded to their mental illness by being overprotective or infantilizing them by hiding sensitive information, thus undermining the individual’s sense of autonomy and independence. Among young persons, familial stigma has been linked to increased self-stigma, for example, Moses (2010) noted that adolescents reported higher self-stigma when parents tended to conceal their child’s mental health issues, whereas parental confidence in their child’s future and ability to control their illness was associated with lower adolescent self-stigma.

For Asian American young adults, family stigma of mental health and loss of face are predominant concerns that impact their decisions to seek mental health services (Leong et al., 2011). Since mental health issues are frequently attributed to a weakness of character in Asian cultures, individuals may fear bringing shame not only upon themselves, but also upon their families, especially if mental health issues affect academic or career success (Yang et al., 2007). Lee et al. (2009) found that because of the fear of being negatively labeled, Asian American young adults tend to conceal their problems from others, neglect or deny their mental distress and not engage in help-seeking. Augsberger et al. (2015) found that young Asian adult women reported family and community stigma as reasons for not seeking mental health services despite acknowledging their need. Among Asian Americans, stigma by close others was associated with self-stigma and linked to negative attitudes towards seeking mental health services or delays in seeking help (e.g., Cheng et al., 2013). In this way, parental messages about mental health stigma and concerns regarding loss of face may deter engagement in professional mental health services among Asian American children and young persons (Leong & Lau, 2001).

## A Need for Empirical Measures of Parental Mental Health Socialization

Despite the central role parental mental health socialization plays in shaping youth mental health beliefs and responses to distress, the advancement of research on this construct has been limited by a lack of existing measures and tools. To date, empirical studies on parental mental health socialization have been largely qualitative in nature, signaling a need for the development of survey measures that capture theoretically identified multidimensional pathways of parental mental health socialization.

In response to this gap, for this current study, we developed and tested the Parent Mental Health Socialization – Parent Response (PMHS-PR) scale, a measure that focuses specifically on the parental mental health socialization pathway of how parents *respond* to youth mental distress. Prior studies have identified parental messages in response to youth distress such as emphasizing relying on the self, stigmatizing mental health problems, dismissing or minimizing youth distress, parental silence, enduring distress and encouraging youth to move or fix their problems (Yasui et al., 2017, 2023 ). However, it remains to be empirically tested whether these parental socialization messages cohere under primary parental mental health socialization pathways (i.e., parental responses to youth mental health) or whether they are distinct avenues of socialization.

To examine the underlying factor structure of the multidimensional construct of parental mental health socialization, we applied the following multidimensional modeling frameworks to test the PMHS-R’s structural validity: (a) a correlated model, (b) higher order model, and (c) bifactor model. The testing of these different model structures was employed to determine whether parental responses in mental health socialization can be conceptualized as a distinct broadband construct that consists of multiple specific narrowband constructs that illustrate parental responses in mental health socialization (i.e., bifactor model), or whether socialization pathways represent constructs that are conceptually related but independent of one another (i.e., correlated model). Given that parental messages to youth distress from prior studies reflected varied culturally anchored responses to youth distress, we also considered a higher order model to determine whether the general construct was mediated by the domain specific factors (e.g., parental mental health stigma; cultural coping).

Specifically, using data from an original study examining mental health beliefs and help-seeking attitudes among East and South-east Asian American young adults, in this study, we (1) developed and tested a new measure of parental mental health socialization, (2) examined the reliability and validity of PMHS-PR, and (3) examined the interrelations between parental mental health socialization processes and Asian American young adults’ stigma beliefs and mental health outcomes.

## Method

### Study Procedures for the Original Study

The current study used data from a larger original study aimed at examining mental health beliefs and approaches to help-seeking among East Asian and South-East Asian American young adults (Yasui et al., 2017, 2023). The study procedures for the original study involved two phases: (a) an exploratory phase of learning mental health beliefs and help-seeking behaviors of Asian Americans via focus groups with 272 self-identifying East Asian and South-East Asian American community members, 47 providers serving Asian American communities and 45 clients utilizing services for mental health, and (b) survey development and testing phase. Eligibility for study participation included the following: (a) self-identifies as East or South-East Asian, (b) between the ages of 12 to 17 for youth and above 18 years old for adults. Participants were recruited through partnerships with local community organizations in Asian American communities and churches as well as student organizations on college and community college campuses. Each focus group was 1.5–2 h in length and consisted of 5–12 people per group, and was conducted by bilingual and bicultural research staff. Participants were compensated for their participation.

The second phase of the original study involved survey development and testing. Items for the surveys were developed from the qualitative data derived from the focus groups in the exploratory phase then tested on a sample of 486 Asian American young adults. Recruitment of participants followed the procedure in the first phase, and all consenting participants completed a two-part online survey that took 60–90 min each and were compensated for their time (Yasui et al., 2017, 2023). All study procedures for the original study were approved by the Institutional Review Board of the university.

### Recruitment of Participants

Participants were recruited through partnerships with local Asian American community organizations, churches, and Asian American student organizations in colleges and community colleges. Participants were eligible for the survey study if they (a) self-identified as Asian American, (b) between the ages of 18–39, and (c) are 1.5 or 2nd generation (Yasui et al., 2017, 2023 ).

### Participants

This current study examined survey data from 486 Asian American young adults from Phase 2 of the original study described above. The sample included 223 male participants (45.88%) and 263 female participants (54.12%) with a mean age of 21.12 years (SD 5.51). Most participants were U.S. born (89. 80%). In terms of ethnicity, 263 participants identified as Chinese American (54.12%) and 223 participants identified as Lao American (45.88%). For the purposes of this study, this sample was randomly split into two: the first for conducting exploratory factor analyses, and the second for confirmatory factory analyses. Sample characteristics of the 2 randomly split samples are shown in Table 1. The 2 samples were similar in terms of sex, age, nativity, and ethnicity.

**Table 1:** Descriptives for study variables

	Sub-sample 1	Sub-sample2	t / Chi
Sample size, n	243	243	
Sex, n (%)			
Male	116 (47.74)	107 (44.03)	0.6712 (*p* = 0.413)
Female	127 (52.26)	136 (55.97)	
Age in years (SD)	23.06 (5.61)	23.18 (5.41)	0.2304 (*p* = 0.8179)
Nativity, n (%)			
U.S. born	191 (78.60)	198 (81.48)	0.6311 (*p* = 0.427)
Foreign born	52 (21.40)	45 (18.52)	
Ethnicity, n (%)			
Chinese American	128 (52.67)	135 (55.56)	0.4060 (*p* = 0.524)
Lao American	115 (47.33)	108 (44.44)	

## Measures

### Development of a Measure of Parental Messages of Mental Health Socialization

Our new measure of parental mental health socialization was developed as part of the original study that examines barriers to treatment engagement among Asian American youth and families. The focus group data from phase 1 of the original study was analyzed using open coding and line by line coding to identify salient codes and themes, that were reviewed and refined into a codebook for later qualitative analyses (Yasui et al. 2017, 2023). The codes developed for the original study served as the basis for preliminary items for the new measure. These preliminary items were then reviewed by experts in Asian American mental health and stakeholders from the Asian American communities. The resulting measure assessed 5 domains of parental mental health socialization that included parental messages on stigma, dismissal or minimization of youth distress, hiding one’s mental health, pursuing success as a solution to distress and parental support. The items were assessed using a 5-point Likert Scale, e.g. (1) “Not at all” (2) “Not much” (3) “Moderately” (4) “Much” and (5) “Very much.” The scale and corresponding items are provided in Table 2.

**Table 2:** Exploratory factor analysis (PAF) with sample 1

Items	Parental stigma toward youth mental health	Parental responsiveness to youth mental health	Parental endorsement of enduring and overcoming distress	Parental cautioning of sharing mental health	Parental silence to mental health
Avoid me or ignore me	**0.81**	-0.03	0.03	0.08	0.18
Treat me poorly because of my problem	**0.85**	-0.01	-0.04	0.04	0.15
Exclude me from community events or social gatherings	**0.84**	0.00	-0.07	-0.17	-0.06
Talk behind my back	**0.54**	-0.07	0.15	0.01	0.02
Hide me from others	**0.88**	-0.09	-0.01	-0.06	0.00
Scold me	**0.71**	-0.18	0.16	0.10	0.10
Force me to stay away from other family members	**0.84**	0.04	-0.01	-0.16	0.02
Keep me home	**0.74**	-0.01	-0.03	-0.08	0.03
Reject me	**0.93**	0.01	-0.01	0.03	0.07
Shame me	**0.82**	-0.11	0.10	0.03	0.03
Treat me as crazy or incapable.	**0.80**	-0.03	0.06	-0.04	0.08
Try to send me away from home for treatment	**0.62**	0.26	-0.03	-0.21	-0.13
Be ashamed of my problem	**0.60**	-0.10	0.13	-0.14	0.03
Blame me for the problem	**0.48**	-0.23	0.27	-0.03	0.10
Told me to go out with people to fix my problems	0.18	**0.40**	0.21	0.02	-0.11
Feel sorry and be sympathetic to me	-0.14	**0.71**	-0.10	-0.04	0.08
Be more willing to help me	-0.18	**0.79**	-0.07	0.02	0.07
Will do more to support me in my daily life activities	-0.08	**0.62**	0.10	-0.07	-0.07
Seek help from others	0.19	**0.56**	-0.08	0.03	-0.20
Encourage me to take care of my mental and emotional health needs	-0.11	**0.74**	-0.12	-0.03	0.06
Encourage me to share my feelings with others	-0.03	**0.79**	-0.09	0.15	0.07
Often ask how I feel	-0.04	**0.73**	-0.04	0.15	-0.03
Listen to me talk about my problems	-0.04	**0.69**	-0.07	0.16	-0.08
Talk to me about mental health and emotional health	0.08	**0.68**	-0.10	0.13	-0.14
Tell me to be grateful for the family sacrifices when I complain about my problems	0.00	-0.19	**0.80**	0.13	0.02
Tell me to tough it out when I say I am experiencing significant distress	-0.02	-0.08	**0.68**	-0.02	0.17
Tell me that I am overreacting or exaggerating my mental health struggles	0.20	-0.17	**0.50**	-0.09	0.08
Tell me to control my problems on my own	0.27	-0.12	**0.55**	-0.02	0.05
Tell me to be grateful for the good and happy life I have instead of being distressed	0.05	-0.06	**0.73**	0.05	0.07
Tell me that I am acting like American kids when I complain about my problems	0.16	-0.01	**0.58**	-0.16	-0.03
Remind me of my ethnic heritage when I complain about how hard things are for me	0.04	-0.03	**0.75**	0.03	-0.01
Tell me that my problems were nothing compared to the difficulties they went through	-0.03	-0.10	**0.76**	0.02	0.00
Tell me that I am too “weak” if I have emotional or mental health struggles	0.34	-0.20	**0.55**	0.00	-0.10
When I am feeling down or upset, they have told me to go do something	0.00	0.17	**0.40**	-0.14	-0.09
Tell me to solve my emotional or mental health struggles on my own	0.15	0.03	**0.41**	-0.12	0.05
Tell me that as long as I am rich or successful, emotional or mental health struggles will not be an issue	0.08	-0.08	**0.52**	-0.16	0.16
Tell me to just focus on my education	0.03	0.02	**0.68**	-0.16	0.13
Tell me to focus on working or studying hard because success is the solution for emotional or mental health struggle	-0.06	-0.04	**0.63**	-0.21	0.09
Remind me of the importance of working hard and achieving goals when I am upset or down	-0.10	0.10	**0.70**	-0.13	0.09
Tell me to not share my emotional or mental health struggles with others	0.15	-0.13	0.14	**-0.63**	0.05
Tell me to keep my personal issues within the family	0.00	-0.21	0.19	**-0.69**	0.04
Tell me to keep my emotional or mental health struggles a secret from siblings or close family members	0.30	-0.04	0.07	**-0.60**	0.06
Tell me to not talk about emotional or mental health struggles with friends or people in the community	0.05	-0.13	0.03	**-0.79**	0.08
They never bring up or talk about my problems	0.15	0.02	0.10	-0.01	**0.74**
No one in my family talks about my problems	0.14	0.07	0.05	-0.09	**0.82**
They do not know about any of my emotional or mental health struggles	0.22	-0.05	0.18	-0.05	**0.40**

#### Parental stigma towards youth

This scale measures youth beliefs regarding parents’ stigmatizing responses to their mental distress including scolding, hiding, blaming, shaming or treating them in negative ways. The 14-item scale reported an internal consistency of 0.97.

#### Hiding mental health from others

The scale on courtesy stigma assesses youth beliefs about parents’ fears of family stigma due to the youth’s mental distress. The scale consists of 4 items and has an internal consistency of 0.92.

#### Enduring and overcoming distress

This 15-item scale measured parental responses to youth distress that conveyed a range of messages, including messages that endorsed enduring distress by focusing on success, pragmatic solutions or “fixes” such as encouraging youths to solve their own problems (e.g. telling them “to go do something”) instead of dwelling on distress. Internal consistency for this scale was 0.94.

#### Parental silence

This 3-item scale captures parental silence in response to youth’s distress that includes both the lack of mention and lack of parental awareness or knowledge of the youth’s distress. This scale had an internal consistency of 0.80.

#### Parental supportiveness in response to youth distress

 Parental responses to youth distress also included supportive responses. This 5-item subscale captured parental responses such as encouraging youth to share their distress, checking in on youth well-being, and having conversations about mental and emotional well-being. The subscale reported an internal consistency of 0.91.

#### Mental health stigma

Stigma towards mental health was assessed by the Stigmatizing Attitudes-Believability (SAB), which is an 8-item self-report questionnaire that assesses public stigma, specifically attitudes toward people with psychological disorders (Masuda et al., 2009). Responses are indicated on a 7-point Likert scale ranging from 1 (not at all believable) to 7 (completely believable). The total score ranges from 8 to 56 and is obtained by summing items, with higher scores indicating higher stigma. Internal consistency for this measure for the sample was 0.87.

### Young Adult Mental Health

#### Depression and somatic symptoms

Young adult depression and somatic symptoms were assessed using the Depression and Somatic symptom scale (DSSS: Hung et al., 2006). The DSSS has two subscales, the depression subscale which consists of 7 items and a somatic subscale which includes 8 items. Internal consistency for the sample was as follows: 0.86 for the depression subscale and 0.86 for the somatic subscale.

### Covariates

Covariates included the following demographic variables (age, gender, household income, and education). Gender was dummy-coded with male as a reference and household income was recategorized into dummy-code with ‘less than $15,000 year’ as a reference.

### Analytic Plan

A split-sample approach was used to examine the multidimensional process of parental mental health socialization (Boateng et al., 2018). Participants with complete data on all scales were included in study. The dataset was split randomly using the split sample code in STATA that allows for a 50/50 split (Stata Corp, 2021). The split samples for Chinese and Lao were equivalent in terms of all of the main study variables resulting in 483 observations in total, 243 for the Exploratory Factor Analysis (EFA) and 243 for the Confirmatory Factor Analysis (CFA).

Next, we employed an item removal strategy suggested by Güvendir and Özkan (2022). Specifically, when an item cross-loaded on two or more factors, the disparity between their respective factor loadings was examined, and when this was below 0.10, the item was removed. A sequential process of item removal was applied, starting with the item with the smallest difference in loadings across factors and subsequently followed until the difference in factor loadings of the item surpassed 0.10. In this way, items that cross loaded onto two or more factors with a loading difference of ≤ 0.10 were first identified, then removed, and we re-ran the processes of extraction with the item removed. Items were retained if they had factor loadings of 0.40 or greater, had low uniqueness (< 0.5), and were conceptually necessary based on face and content validity.

Following the removal of cross-loaded items, we conducted parallel analysis in order to be parsimonious in determining the number of factors to retain. Parallel analysis uses a Monte Carlo simulation to compare the eigenvalues from data prior to rotation with eigenvalues obtained from a random matrix that has the same number of variables and sample size with 100 replications (Velicer et al., 2000). Means and standard deviations of the replicated eigenvalues are calculated for each component, and the value for the 95th percentile is obtained, which serves as the standard to which the eigenvalues of each component from the research data are compared. A component was retained if we found that the eigenvalue was beyond the 95th percentile (i.e., more than 95% confidence that it is not obtained at random). In our data, five factors had eigenvalues greater than those generated by the random data.

EFA was then conducted with five factors using principal axis factoring (PAF) and the oblimin rotation was used to allow factors to correlate for Sample 1 (*n* = 243). The appropriateness of running factor analysis was assessed with the Kaiser-Meyer-Oklin Measure of Sampling Adequacy (KMO) ≥ 0.6 and with Bartlett’s Test of Sphericity being significant (*p* < 0.05) (Barlett, 1954; Kaiser, 1974; Field, 2013). The Kaiser-Meyer-Olkin measures of sampling adequacy was 0.947 and the Bartlett’s test of sphericity was *p* < 0.001, supporting the appropriateness of conducting EFA on our sample.

Next, we conducted a series of CFA with five factors with Sample 2 (*n* = 243) to confirm the factor structure of the measure of parental mental health socialization derived in Sample 1. We allowed only primary factor loadings in order to achieve a simple structure for the CFA. In addition, for all CFAs, we examined model fit indices to determine the nature of items assessing the five domains of parental mental health socialization. Following the results derived from the EFA, we tested the adequacy of the fit of alternate factor structures to determine the advantage of bifactor models over other multidimensional structures. The factor structures tested included a correlated 5 factor model, a higher-order factor model, and a bifactor model. For the correlated 5-factor model, the seven factors were specified based on the EFA results, and each item was specified to load on one of the five factors. In the higher-order structure model, items that loaded onto the five lower order factors that shared a common source of variance were identified for the higher order factor. For the bifactor model, we had all of the items load onto a general factor, and residual variance was explained by the five orthogonal first-order factors.

To examine the models’ goodness of fit, we considered the chi-squared value for the overall model fit, the comparative fit index (CFI), the Tucker–Lewis index (TLI), the root mean square error of approximation (RMSEA), and the standardized root mean square residual (SRMR). Hu and Bentler (1999) suggested a cutoff value of 0.90 and 0.95 as a reasonable and close fit for CFI and TLI, for SRMR a cutoff value of 0.08 for a reasonable fit and for the RMSEA < 0.05 as a good fit, between 0.05 and 0.10 a fair to mediocre fit (MacCallum et al., 1996).

Further, to test the measurement fit for each subscale, the reliability, descriptive statistics and item-total correlation were examined (Nunnally & Bernstein, 1994). Last, to test the validity of the new measure of parent mental health socialization, bivariate correlations were conducted to determine the interrelations with and existing measures of mental health symptoms, and stigma attitudes towards mental illness.

## Results

### Descriptives

Descriptive statistics for the study variables are reported in Table 1. Of the 486 participants, the sample included 223 male participants (45.88%), and 263 female participants (54.12%) with a mean age of 21.12 years (SD 5.51). Most participants were U.S. born (389, 80%). In terms of ethnicity, 263 participants were identified as Chinese descendants (54.12%) and 223 participants were identified as Laotian descendants (45.88%). Sample 1 consisted of 243 participants (128 Chinese American, 115 Lao American) and 47.74% were male and Sample 2 consisted of 243 participants (135 Chinese American, 108 Lao American) and 44.03% were male.

### Exploratory Factor Analysis

Principal axis factoring (PAF) was initially performed on Sample 1 with all 63 items (*n* = 243). An iterative process was used where items that cross-loaded onto two or more factors with a loading difference of less than 0.10 were first identified, then removed, followed by rerunning the processes of extraction. This procedure eliminated 20 items, resulting in a refined item pool of 43. Parallel analysis on this adjusted pool of items indicated a five-factor solution. We then employed PAF with oblique rotation, which allowed for the accommodation of expected correlations among factors, extracting five factors. The factorability of the PMHS-PR was examined using two criteria: (1) the mean of the Kaiser-Meyer-Olkin measure of sampling adequacy which was 0.95 and well above the recommended value of 0.60 (Cerny & Kaiser, 1977), and (2) Bartlett’s test of sphericity, which was significant (χ2 (1035) = 10035.22, *p* < 0.01), suggesting that these items were intercorrelated and suitable for factor analysis. The PAF with direct oblimin rotation revealed 5 components with eigenvalues ≥ 1 (20.03, 4.77, 3.00, 1.48, 1.14, 1.05), accounting for 66.72% of the variance. A total of 14 items loaded on first component (*parental stigma towards youth*), 10 items loaded on the second component (*parental supportiveness*), 15 items on the third component (*endure and overcome distress*), 4 items on component 4 (*hiding mental health from others*), and 3 items on the 5th component (*parental silence*). The components and corresponding items are presented in Table 2.

### Confirmatory Factor Analyses

A series of CFAs were conducted on Sample 2 (*n* = 243) including the five correlated first order factor structure, a higher order factor structure, and a bifactor structure (see Table 3 for summary of fit indices). For the correlated CFA, results showed that the model had a poor model fit with the data, χ2 (979) = 2711.18, *p* < 0.05; RMSEA = 0.09; CFI = 0.81). We also conducted a higher order factor structure with components 1 (parental stigma), 3 (minimizing youth distress) and 4 (success as a solution loading on a higher order construct, which indicated a poor model fit, χ^2 (983) = 2722.70, *p* < 0.05; RMSEA = 0.09 CFI = 0.81). Finally we conducted a bifactor model that indicated a good fit, χ2 (932) = 1832. 82, *p* < 0.05; RMSEA = 0.06; CFI = 0.90). These findings suggested that this newly developed measure of parent response in mental health socialization should not be considered as comprising of a single general factor of mental health socialization. Instead, the results showed that the confirmatory bifactor structure was a more appropriate fit with the data, suggesting that the measure captures a domain-general factor on parental mental health socialization and seven domain specific factors that assess unique parental messages of mental health.

**Table 3:** Summary of the fit indices across three models for parental responses in mental health socialization

Model	χ²	df	CFI	RMSEA (90% CI)	AIC	BIC
Five-Factor Correlated Model	2711.18	979	0.809	0.085	27984.09	28501.06
Higher Order Factor Model	2722.70	983	0.808	0.085	27987.60	28490.60
Bifactor Model	1832. 82	932	0.901	0.063	27199.72	27880.87

### Reliability and Correlations

Internal consistency reliability for all five scales was adequate to excellent ranging from 0.73 to 0.96 for Sample 2. In addition, zero order correlations were between the related constructs of parent mental health socialization were examined (see Table 4). Four of the five factors showed strong positive correlations, suggesting the interrelatedness of these pathways of parental mental health socialization. As expected, *parental supportiveness* showed negative associations with other four factors, indicating a distinct parental response to youth distress.

**Table 4:** Correlations between the factors, stigmatizing attitudes, and young adult mental distress symptoms (Sample 2)

	(1)	(2)	(3)	(4)	(5)	(6)	(7)	(8)
(1) Parental stigma towards youth	1.00							
(2) Parental supportiveness	− 0.26*	1.00						
(3) Enduring and overcoming distress	0.72**	− 0.37**	1.00					
(4) Hiding mental distress from others	0.57**	− 0.17*	0.62**	1.00				
(5) Parental Silence	0.48*	− 0.45**	0.57**	0.37**	1.00			
(6) Stigmatizing Attitudes Towards Mental Illness	0.29**	0.19*	0.16*	0.11	0.08	1.00		
(7) Depressive Symptoms	0.22**	− 0.04	0.33**	0.22**	0.20**	0.10	1.00	
(8) Somatic Symptoms	0.17**	− 0.01	0.31**	0.24**	0.22**	0.04	0.77**	1.00
Mean (SD)	2.19 (0.93)	3.28 (0.73)	3.08 (0.94)	2.73 (1.08)	2.98 (0.94)	2.70 (0.49)	1.95 (0.81)	2.21 (0.79)
Internal Consistency (Sample 2)	0.96	0.86	0.95	0.91	0.73	0.87	0.86	0.86

* shows significance at the 0.05 level

To examine concurrent validity, the newly developed scales were correlated with measures of mental health stigma and youth mental health outcomes. Stigmatizing attitudes towards mental illness were positively correlated with *parental stigma towards youth* and *endure and overcome distress* and negatively associated with *parental supportiveness.* Interestingly, *hiding mental health from others* and *parental silence* were unrelated to young adult stigma attitudes towards mental health. Young adult mental health symptoms were positively linked to *parental stigma towards youth*,* endure and overcome*, *hiding mental health from others* and *parental silence*. *Parental supportiveness* was unrelated to neither depressive or somatic symptoms.

## Discussion

The purpose of this study was to develop the Parent Mental Health Socialization – Parent Response (PMHS-PR) scale, a new measure of parental mental health socialization and to evaluate its psychometric properties in a sample of Asian American young adults. Using randomly split samples, EFA and CFAs were conducted separately. The EFA favored a five-factor solution, which explained over 67% of the variance. The five factors being derived revealed that parents conveyed distinct messages in response to youth mental distress: *parental stigma towards youth*, *parental endorsement of enduring* *and overcoming* *distress*, *hiding mental health from others*, *parental silence*, and *parental supportiveness*. Results for the CFAs confirmed that the bifactor structure model yielded the best fit, providing empirical support for a general, common construct as well as five domain-specific factors of parental responses in mental health socialization. All five scales showed acceptable internal consistency and indicated acceptable concurrent validity with somatic symptoms, and all except for parental supportiveness showed significant positive correlations with young adult depressive and somatic symptoms. Three of the five scales (*parental stigma towards youth*, *parental endorsement of enduring* *and overcoming* *distress*, *parental supportiveness*) also had positive associations with stigmatizing attitudes towards mental health, suggesting their validity in capturing salient processes of mental health socialization. Overall, the EFA/CFA confirmed the multidimensionality of processes of a broad latent construct of parental socialization of mental health.

The current study illustrates that parental responses to youth distress encompass a set of pathways in parental mental health socialization. While cohering as one general factor, the distinctiveness of the five factors indicates that parents respond with messages of mental health in a variety of ways – which may have differing effects on the youth. The first factor, *parental stigma toward youth*, encapsulates parental stigma responses, whether it be directed toward the youth themselves, or fearing the effects of stigma from others (e.g., parental stigma towards youth, hiding mental health from others). These parental responses are reflective of the stigmatized views of mental health that are documented among Asian Americans (e.g., Hsiao et al., 2006; Lee et al., 2009), and signal the implications of parental mental health stigma on familial processes and youth mental health outcome. While existing literature has noted the link between stigma views and discriminatory behaviors from others (e.g., Brohan et al., 2013), this study is one of the first that has examined how stigma manifests within the family, and particularly how it is received. From our findings, youths who endorsed receiving messages of stigma from parents reported more elevated levels of depressive and somatic symptoms which aligns with studies reporting the negative psychological effects of stigma (Lee et al., 2009).

The factor, *enduring and overcoming distress* reflects the Confucian value, *chi de ku zhong ku*,* fang wei ren shang ren*, describes the virtue of tolerating hardship to increase one’s stature (Shek, 2004). Parental messages included those that exhorted youth to overcome their distress through gratitude, problem-solving, or pursuing success. Parents may respond with these messages of enduring and overcoming distress because Asian cultural views tend to attribute mental distress to weakness of character, willpower, or lack of self -control, all of which will likely be met with significant social disapproval (Hsiao et al., 2006). While correlational, the effect of these messages, however, appear to be negative — our findings link messages of endurance with more severe youth mental distress. Young adults who receive messages of enduring or overcoming distress may feel unheard, worry that their mental health problems will be a burden to family members, or fear that their family will be stigmatized due to their distress (Anyon et al., 2013).

The factor, *hiding mental health from others* described parental responses that discouraged youths from discussing their mental distress with others. Parental messages included keeping information on the youth’s distress within the family, preventing its disclosure to others, including friends, people in the community, and even other family members. In Asian cultures where Confucian principles are emphasized, mental health problems are often viewed as a moral or social failing, and hence perceived as a source of shame and loss of face (Hsiao et al., 2006; Zane & Yeh, 2002 ). Parents may anticipate potential stigma and discrimination from others, and thus caution their youths to be discreet about their mental health problems. Studies suggest that fears of shame and stigma can result in family members hiding the mental health problems of their loved ones (Chong et al., 2007) and even reject or delay seeking mental health services (Ling et al., 2014). Moreover, our findings suggest a link pertaining to receiving messages that endorsed hiding mental health problems from others, where receipt of such messages was associated with higher reported somatic and depressive symptoms.

*Parental silence* emerged as a factor that described the absence of mention or lack of acknowledgement or awareness of the youth’s mental distress. Parents may engage in silence due to a variety of reasons including the denial or dismissal of youth mental distress, and lack of awareness of youth distress (Yasui et al., 2023 ). By responding in silence, parents may reinforce cultural norms on the suppression of emotion (Yeh & Inose, 2002) as well as the taboo of openly acknowledging or discussing psychological or emotional problems (Yong & McCallion, 2004). On the other hand, parental silence may also reflect the parents’ lack of awareness or obliviousness to youth mental distress that stems from differential understandings and conceptualizations of mental health problems. For example, if the parents’ conceptualizations of mental distress are based on frameworks of traditional Chinese medicine (which emphasize the interconnectedness of mind and body), they may expect youths to report physical rather than psychological symptoms during distress, thereby disregarding or overlooking signs of youth distress (Choi et al., 2016). For young adults in our study, parental silence was associated with higher levels of depressive and somatic symptoms, which may suggest that the absence of an open communication channel regarding youth distress may indicate an association between parental silence and youth mental health symptoms.

*Parental supportiveness* captured a range of responses including listening or asking about the youth’s distress, helping youth by providing practical support, and encouraging self-care. Existing literature indicates that parental support is vital to the healthy development of youth and youth perceptions of parental support are associated with youth’s physical health status (Patten et al., 1997). Thus, parental facilitation of open communication that encourages youths to share their distress allows children to develop a healthy understanding of their problems in context and normalizes their fears (Garbarino et al., 1992). As expected, parental supportiveness was negatively associated with other mental health socialization factors, and no association was found with youth depressive or somatic symptoms.

### Implications for Use

The current study suggests that the PMHS-PR can be a useful tool for both researchers and clinicians understand the various ways parents socialize their children with respect to mental health and healing. Our empirical findings in the EFA and, in particular, the bifactor CFA, suggests that this measure captures the broadband construct of parental responses in mental health socialization that is manifested through multiple pathways, with differential effects on young adult mental health.

The five factors in the PMHS-PR showed the range of socialization pathways of mental health from stigma towards youth, parental silence, to parental supportiveness. Researchers and clinicians who use the PMHS-PR may find having subscales that assess specific pathways of mental health socialization to be useful, especially in discerning messages that either facilitate or impede health development in youths. Understanding how the passing down of parental messages occurs is likely to be particularly helpful in intervention, as it can guide clinicians to determine targets for treatment. Further, information gained from the PMHS-R may be used to inform the development and implementation of culturally attuned mental health literacy or psychoeducational programs for Asian American youth and young adults. This may be particularly important given the low mental health literacy rates among Asian American populations (Yasui et al., 2017). While preliminary, our findings highlight salience of parental mental health socialization as a construct and its importance in understanding the varied ways in which parents convey messages regarding mental health, particularly when responding to young adults’ experiences of distress.

### Limitations and Future Directions

Although the bifactor structure in our study revealed the multidimensionality of parental mental health socialization, the PMHS-PR was not developed to exhaustively capture processes of parental mental health socialization. In fact, our measure focused specifically on addressing parental responses in mental health socialization. Thus, the measure does not capture other domains of mental health socialization such as the passing down of parental views of mental health or help-seeking behaviors. For example, Yasui et al. (2023) identified parental conceptualizations of mental distress and parental responses to youth distress as central processes of parental mental health socialization, highlighting the need for future studies to identify and develop measures that appropriately capture other domains of mental health socialization. Additionally, the current measure assessed only youth reports of receiving parental messages of mental health socialization. It will be crucial for measures of parent reported mental health socialization to be developed to identify convergences and discrepancies in how parents engage in conversations about mental health within the familial context. Furthermore, our study examined parental mental health socialization processes within Asian American young adults – therefore future studies will be required to examine whether the measure can be used for families of other ethnicities and races, given that understandings of mental health vary cross-culturally. Finally, our data is cross-sectional, which limits our understanding of the longitudinal effects of parental messages of mental health on mental health outcomes in young adults. Future studies should examine the predictive effects of mental health socialization on young adult mental health as well as how they may shape perceptions and attitudes towards mental health.

Nonetheless, despite these limitations, our study demonstrates the importance of understanding parental mental health socialization as a salient process within the family context and that the PMHS-PR is an effective measure that allows for assessing the specific pathways of these socialization processes among Asian American families. Understanding how mental health is perceived and communicated within Asian American families will be crucial in identifying how to better inform interventions that address the mental health needs of Asian American and immigrant youth and families. It is our hope that as one of the few measures of parental mental health socialization, the PMHS-PR will be used in additional studies to further expand our understanding of the socialization process of mental health across Asian American families and other culturally diverse communities, both in the United States and globally.

## Supplementary Information

Below is the link to the electronic supplementary material.

Supplementary Material 1
