# Supplementary material for: What Parents Say and do: Parental Responses to Asian American Young Adult Mental Health and Help Seeking
Source: J Child Fam Stud. 2026 Jan 29;35(3):767–81. doi: 10.1007/s10826-026-03259-4 (PMC12953297; doi:10.1007/s10826-026-03259-4)
Supplement: Supplementary file 1 — Supplementary Material 1 [file 10826_2026_3259_MOESM1_ESM.docx]

**Funding**

This study was funded by the Agency for Healthcare Quality and Research (AHRQ) Grant # AHRQ: 5K12 HS023007

**Compliance with Ethical Standards**

**Conflict of Interest** The authors declare no competing interests.

**Ethical Approval** All procedures performed in studies involving human participants were in accordance with the ethical standards of the ethics committee of the University of Chicago. All procedures performed in the study were in accordance with the 1964 Helsinki declaration and its later amendments or comparable ethical standards.

**Informed Consent**

Informed consent was obtained from all participants in the study
